# Prevalence and risk factors of acute respiratory infection and diarrhea among children under 5 years old in low-middle wealth household, Indonesia

**DOI:** 10.1186/s40249-025-01286-9

**Published:** 2025-02-27

**Authors:** Tri Bayu Purnama, Keita Wagatsuma, Reiko Saito

**Affiliations:** 1 Division of International Health (Public Health), Graduate School of Medical and Dental Sciences, Niigata University, 1-757 Asahimachi Dori, Chuo-Ku, Niigata, 951-8510 Japan; 2 Faculty of Public Health, Universitas Islam Negeri Sumatera Utara, Medan, Indonesia; 3 Institute for Research Administration, Niigata University, Niigata, Japan

**Keywords:** Respiratory tract infections, Diarrhea, Malnutrition, Social protection, Indonesia

## Abstract

**Background:**

Acute respiratory infection (ARI) and diarrhea remain critical public health concerns. In Indonesia, various interventions have been implemented to reduce the prevalence of ARIs and diarrhea among children in low- and middle-income households. Hence, the absence of detailed data on the prevalence of ARIs and diarrhea among children under five in low- and middle-income households in Indonesia restricts the formulation of targeted health interventions and policies. This study sought to evaluate the prevalence of ARI and diarrhea while examining modifiable risk factors related to malnutrition, sanitation, and social protection characteristics in Indonesia.

**Methods:**

This study utilized a cross-sectional design based on data from the Nutrition Status Survey 2022 covering 514 districts/cities in Indonesia. It analyzed 289,631 children under five years out of 334,848 households with low and middle wealth indices. Multivariable binary logistic regression analysis was employed to calculate the risk associated with cases of ARI and diarrhea.

**Results:**

The prevalence of ARI and diarrhea among low- and middle-wealth households were 5.7% and 6.0%, respectively, with infants under six months being the most vulnerable group to these infections, including malnutrition. The most significant risk factors for ARI and diarrhea are unclean cooking fuel [adjusted odds ratio (a*OR*) = 1.53, 95% *CI* 1.47–1.60] and shared toilet facilities (a*OR* = 1.45, 95% *CI* 1.38–1.51), with households using shared toilets having 1.45 times higher risk of diarrhea (a*OR* = 1.45, 95% *CI* 1.38–1.51) compared to those with private access. Additionally, households lacking social protection support are also at increased risk for these infections and malnutrition issues.

**Conclusions:**

This study revealed a notable prevalence of ARI and diarrhea among low- and middle-wealth households, particularly affecting infants under six months. Vulnerable children, especially those who were stunted or underweight, and households lacking sanitation and social protection faced heightened risks for these health issues.

**Graphical Abstract:**

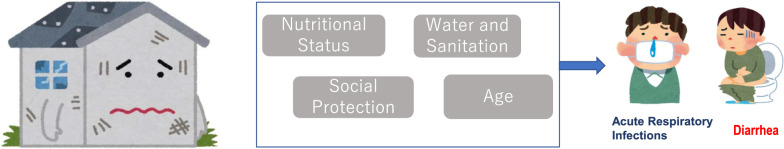

**Supplementary Information:**

The online version contains supplementary material available at 10.1186/s40249-025-01286-9.

## Background

Acute respiratory infection (ARI) and diarrhea remain critical public health concerns, associated with global disease burden [1, 2] and contributing to approximately one-quarter of all mortality among children under the age of five [3]. The socioeconomic impacts on children affected by ARI and diarrhea include developmental delays, malnutrition, and diminished future educational opportunities [3]. These conditions are disproportionately prevalent in developing countries, particularly Southeast Asia, where healthcare access and sanitation infrastructure are limited [1, 2]. In Southeast Asia, the prevalence of diarrhea ranges from 8.39% in the Philippines to 18.21% in Indonesia [4], while ARI episodes are estimated to occur at a rate of 133.9 per 1000 children under five years old [2].

In Indonesia, the Health Survey conducted by the Ministry of Health in 2022 reports an incidence rate of 4.8% for ARIs and 4.9% for diarrhea, with regional incidence rates varying according to geographic and socioeconomic factors [5]. Indeed, co-infections with multiple enteric pathogens substantially contribute to diarrheal disease in children under five, with bacterial and viral agents assuming distinct roles in modulating both disease severity and transmission pathways [6, 7]. Indonesia has adopted the Integrated Management of Childhood Illness (IMCI) approach, which incorporates the treatment of pneumonia and diarrhea within national healthcare services [8]. Despite the significant reduction in the under-five mortality rate, from 58 per 1000 live births in 2007 to 32 per 1000 live births in 2017 [8], limitations in resources, funding, logistics, and equipment have hindered the optimal implementation of the IMCI program [9]. While IMCI was designed to enhance equitable access to childcare services for low-income families, its effectiveness has been constrained by structural challenges, implementation gaps, and insufficient continuity of care across healthcare levels [10]. These findings reinforce the association between lower socioeconomic status, malnutrition, and higher ARI rates, highlighting the need for targeted public health interventions [11], as observed in remote areas of Indonesia where efforts to address malnutrition are linked to high rates of infectious diseases in children under five [12].

An important, yet under-researched factor contributing to ARIs and diarrhea in children under five in Indonesia is the variation in modifiable risk factors across different socioeconomic groups. The Indonesian Ministry of Health has reported that socioeconomic development in eastern Indonesia tends to be lower compared to western Indonesia [13]. This is particularly evident in Eastern Indonesia, where challenges in accessing healthcare—both geographically and economically—contrast sharply with conditions in the western part of the country [14]. Low- and middle-income households in Indonesia often face inadequate access to water, sanitation, and hygiene, including unsafe drinking water, limited sanitation facilities, poor hygienic conditions, and restricted access to healthcare. These factors play a crucial role in the incidence of ARIs [11] and diarrhea among children under five. A pronounced wealth gap persists, where children from the poorest quartile experience more significant morbidity and slower reductions in the prevalence of diarrhea, ARIs, and malaria compared to those from wealthier quartiles [15]. Financial stress experienced by parents is associated with detrimental outcomes for children’s health, including increased risk of undernutrition, impaired cognitive development, and weakened immune responses, all of which contribute to greater vulnerability to diseases [16].

Indonesia continues to experience relatively higher rates of ARI and diarrhea compared to other Southeast Asian countries (e.g., Cambodia, Myanmar, and the Philippines) [1, 2]. Currently, there is limited evidence regarding the prevalence of ARIs and diarrhea in this age group among low-middle wealth household in Indonesia. Using a large-scale Indonesia national survey, this study aimed to quantify the prevalence of ARI and diarrhea among households in the lower to middle range of the relative wealth index and identify risk factors, considering individual and household characteristics.

## Methods

### Study design and setting

**Fig. 1:**
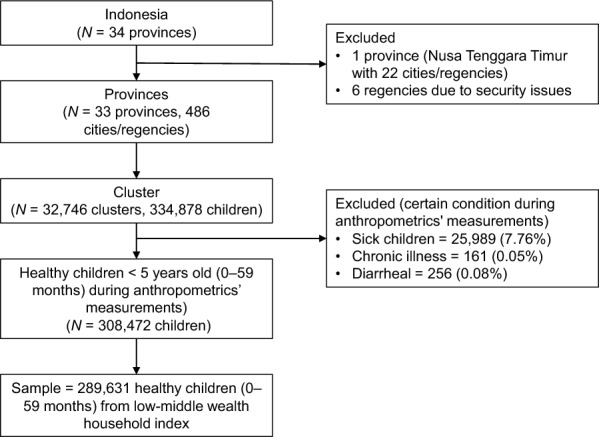
Data procedures

This study utilizes a cross-sectional study design based on data from the 2022 Indonesian National Nutritional Status Survey, conducted by the Ministry of Health of Indonesia. The study relies on this survey as secondary data. The National Ethics Commission determined that the study qualifies for exemption. Data collection for the 2022 survey was carried out by the Ministry of Health of Indonesia, with informed consent obtained through signed agreements. Participants provided written consent, underscoring the voluntary nature of participation and the confidentiality of the collected data. The survey implemented a 2-stage stratified cluster sampling technique to focus on addressing nutritional issues in Indonesia [17]. In the first stage, 34,500 census blocks were randomly selected from a pool of 190,225 census records in the 2020 population census for 514 districts/cities. In the second stage, 10 households with children under five years old were sampled from each census block, resulting in 345,000 households (Fig. 1). The initial dataset comprised 334,848 children under five years of age from 486 districts and cities participating in the nutrition survey between September and November 2022. After data cleaning, only healthy children were retained for further analysis.

### Data procedures and variables

This study focuses on respondents from the backgrounds of low and middle-socioeconomic households to assess the prevalence and risk factors of ARI and diarrhea. To select respondents, the study utilized an approach grounded in the wealth status of households, which assessed living standards through ownership of assets, focusing on relevant indicators of wealth [18]. Asset-based measures facilitate the ranking of households in the absence of consumption data and have been demonstrated to be more stable and less susceptible to temporary fluctuations than consumption data [19]. Principal component analysis was commonly employed to classify the wealth levels of households based on the assets owned by each household [18]. The lowest quartile (quartile 1) represents the poorest households, while the highest quartile (quartile 5) represents the wealthiest households. In this study, only households in quartiles 1 to 3 were included in the final analysis, comprising a total of 289,631 children under five years old.

The primary outcomes of this study were the incidences of ARI and diarrhea. ARI was identified based on symptoms such as fever, a cough lasting less than two weeks, runny nose, and/or sore throat. Diarrhea was defined by the passage of three or more loose or liquid stools per day, excluding cases with blood in the stools. All diagnoses were made by a medical doctor within the month before the survey [17]. The definitions of ARI and diarrhea have been consistently utilized by the Demographic Health Survey from 2014 to 2021 [3]. The nutrition survey data were collected through direct interviews with parents or caregivers and individual anthropometric measurements. Nutritional status was evaluated using standard anthropometric indicators, including *Z*-scores for height-for-age (HA), weight-for-height (WH), and weight-for-age (WA), derived from age, height, and weight.

Household environmental factors were also incorporated into the analysis, such as using residual fuels for cooking (e.g., kerosene, charcoal, firewood), access to improved drinking water, and shared toilet facilities. Improved drinking water was defined based on the World Health Organization’s criteria, encompassing water sources like household connections, public standpipes, boreholes, and rainwater collection systems protected from external contamination, particularly fecal matter [20]. The use of shared toilets was categorized as an environmental exposure factor associated with ARIs and diarrhea [20].

Social protection is defined as households recorded by the government of Indonesia to receive at least one official assistance program, such as the Family Hope Program (Program Keluarga Harapan), Non-Cash Food Assistance Program (Bantuan Pangan Non Tunai), Direct Cash Assistance of the cooking oil support (Bantuan Langsung Tunai Minyak Goreng), Direct Cash Assistance of the Village Funds (Bantuan Langsung Tunai Dana Desa), and the Pre-Employment development support (Kartu Prakerja). These social protection programs are designed to assist low-income households in meeting the essential needs for a dignified life [21].

### Statistical analysis

#### Descriptive statistics

This study constructed wealth index using principal component analysis considered household assets, including gas cylinders (yes/no), washing machines (yes/no), refrigerators (yes/no), air conditioners (yes/no), water heaters (yes/no), landline phones (yes/no), smartphones (yes/no), computers/personal computers (yes/no), motorcycles (yes/no), gold/jewelry (yes/no), boats (yes/no), motorboats (yes/no), cars (yes/no), land/plots(yes/no), and television of at least 32 inches (yes/no). Households were classified into five wealth index quartiles, where a higher quartile position corresponds to an increased wealth index. Additionally, this study calculated height-for-age *Z* score (HA*Z*) for stunting, weight-for-height *Z* score (WH*Z*) for wasting, and weight-for-age *Z* score (WA*Z*) for underweight, with each indicator defined by a *Z* score below –2 standard deviations [22]. The assessments of wealth index and nutritional status, respectively, were conducted using the “*psych*” and “*anthro”* packages in R version 4.3.1, and provided by the R Foundation for Statistical Computing, Vienna, Austria [23, 24]. Descriptive statistics for the various variables were calculated using frequencies and proportions, while the chi-square test was employed to examine statistical differences in proportions. Furthermore, this study utilized digital administrative maps sourced from the Ministry of Health, Indonesia, and employed Quantum Geographic Information System (QGIS) software version 3.32.3-Lima [25] to map ARIs and diarrhea. The natural Jenks breaks optimization method was applied to analyze the spatiotemporal patterns of these health conditions [26].

#### Multivariable binary logistic regression analysis

Univariate and multivariate binary logistic regression was conducted to explore the associations between the dependent (ARI and diarrhea) and independent variables (age, sex, stunting, wasting, underweight, cooking fuel, drinking water, shared toilet used, and region type). Variables that yielded *P* < 0.2 in the logistic regression were included in the multivariable binary logistic regression analysis to estimate the adjusted odds ratio (a*OR*), reflecting the strength of the relationship between the risk factors for ARI and diarrhea. Before the multivariable binary logistic regression analysis, multicollinearity was evaluated using the variance inflation factor (VIF) and a correlation matrix, considering the independent variables. The evaluation of multicollinearity indicated no significant issues (Supplementary material 1). Additionally, the correlation coefficients among the variables were mostly below 0.8, suggesting that collinearity is not expected to pose a problem in this analysis (Supplementary material 2). Furthermore, we evaluated the overall goodness of fit through the Hosmer–Lemeshow tests. The findings revealed that the observed data was more accurately explained by the diarrhea model [X^2^(8) = 9.87, *P* = 0.281] in comparison to the ARIs model [X^2^(8) = 21.17, *P* = 0.007]. We employed multivariable binary logistic regression analysis using R version 4.3.1 (R Foundation for Statistical Computing, Vienna, Austria). In the multivariable binary logistic regression analysis, statistical significance for all analyses was set at 0.05 (two-tailed), with 95% confidence intervals (*CI*s) computed.

### Ethical considerations

This study analyzed data authorized by the Ministry of Health, Indonesia. The epidemiological data used in this research had been anonymized to maintain confidentiality. Ethical clearance was not required because the analysis used publicly available data with no identifiable information. The study complied with the principles of the 2013 revision of the Declaration of Helsinki.

## Results

### Descriptive statistics

A total of 289,361 children under 5 years of age were subjects in this study, originating from lower and middle-wealth households. The prevalence of ARI and diarrhea were 5.7% (16,648/289,361) and 6.0% (17,268/289,361), respectively. Diarrhea cases were more widely distributed across Indonesia than acute respiratory infections (ARIs). However, several regions did not report any ARI cases during the survey period. Interestingly, areas outside of Java, particularly in Sumatra and Papua, showed a higher frequency of ARI reports compared to diarrhea cases with more specifically Asmat Regency showing a particularly high prevalence of both ARIs (49.6%) and diarrhea (44.1%) (Supplementary material 1). Respondents aged 6–59 months were reported more frequently diagnosed with ARI (15,939, 5.5%) and diarrhea (16,753, 5.8%) compared to aged 0–5 months (*P* < 0.001). Of the total study respondents, the proportion of children suffering from ARI and experiencing stunting issues (3849, 1.3%) tended to be higher (*P* < 0.001) compared to those with underweight (2942, 1.0%) and wasting (1427, 0.5%) (Table 1). A similar pattern was shown in children with diarrhea who also experienced stunting (4104, 1.4%), underweight (3298, 1.1%), and wasting (1584, 0.5%). Children with malnutrition problems were associated with ARI and diarrhea issues (*P* < 0.001). In malnutrition conditions, children aged 24–59 months were recorded as the age group with the highest cases of stunting (41,577, 14.4%), underweight (32,596, 11.3%), and wasting (13,448, 4.6%) compared to the age groups of 0–5 and 6–23 months (*P* < 0.001).

**Table 1:** Socio-demographic of acute respiratory infection and diarrhea among relative middle-low wealth household

	Acute respiratory Infections		*P* value	Diarrhea		*P* value
	Yes (%)	No (%)		Yes (%)	No (%)	
Malnutrition						
Stunting (yes)	3849 (1.3)	54,690 (18.9)	< 0.001	4104 (1.4)	54,434 (18.8)	< 0.001
Underweight (yes)	2942 (1.0)	41,927 (14.5)	< 0.001	3298 (1.1)	41,571 (14.4)	< 0.001
Wasting (yes)	1427 (0.5)	19,999 (6.9)	< 0.001	1584 (0.5)	19,842 (6.9)	< 0.001
Age						
0–5 month	709 (0.2)	23,365 (8.1)	< 0.001	515 (0.2)	23,559 (8.1)	< 0.001
6–59 month	15,939 (5.5)	249,618 (86.2)		16,753 (5.8)	248,803 (85.9)	
Sex						
Male	8681 (3.0)	139,896 (48.3)	< 0.01	9348 (3.2)	139,229 (48.1)	< 0.001
Female	7967 (2.8)	133,087 (46.0)		7920 (2.7)	133,133 (46.0)	
Region type						
Urban	8236 (2.8)	138,274 (47.7)	< 0.001	8504 (2.9)	138,005 (47.6)	< 0.001
Rural	8412 (2.9)	134,709 (46.5)		8764 (3.0)	134,357 (46.4)	
Social protection ownership						
No	3670 (1.3)	57,650 (19.9)	< 0.01	4197 (1.4)	57,123 (19.7)	< 0.001
Yes	12,978 (4.5)	215,333 (74.3)		13,071 (4.5)	215,239 (74.3)	
Wealth index						
Poor	15,025 (5.2)	246,998 (85.3)	0.05	15,809 (5.5)	246,213 (85.0)	< 0.001
Middle	1623 (0.6)	25,985 (9.0)		1459 (0.5)	26,149 (9.0)	
Cooking fuel						
Unclean	3772 (1.3)	42,944 (14.8)	< 0.001	2598 (0.9)	44,117 (15.2)	< 0.001
Clean	12,876 (4.4)	230,039 (79.4)		14,670 (5.1)	228,245 (78.8)	
Drinking Water						
Unimproved	6835 (2.4)	107,482 (37.1)	< 0.001	6960 (2.4)	107,356 (37.1)	< 0.001
Improved	9813 (3.4)	165,501 (57.1)		10,308 (3.6)	165,006 (57.0)	
Shared Toilet						
Yes	2021 (0.7)	25,897 (8.9)	< 0.001	2273 (0.8)	25,645 (8.9)	< 0.001
No	14,627 (5.1)	247,086 (85.3)		14,995 (5.2)	246,717 (85.2)	

A significant difference in proportion (*P* < 0.001) was found among children under five from households using unclean cooking fuel who experience ARI (3772, 1.3%). Furthermore, children from households with unimproved drinking water (6835, 2.4%) and shared toilet facilities (2273, 0.8%) also showed a significant difference (*P* < 0.001) in relation to diarrhea cases. These disparities extend to the issues of unclean cooking fuel, unimproved drinking water, and shared toilet usage concerning malnutrition indicators such as stunting, underweight, and wasting (*P* < 0.001).

A total of 3670 (1.3%) and 4197 (1.4%) respondents who experienced ARI and diarrhea (*P* < 0.001), respectively, did not have social protection (governmental support encompasses both financial assistance and non-financial assistance), including those who were stunted (15,098, 5.2%), underweight (11,458, 4.0%), and wasted (4,977, 1.7%) (*P* < 0.001) (Table 2). Respondents with social protection were more often found among those with malnutrition problems compared to those diagnosed with ARI (*P* < 0.01) and diarrhea (*P* < 0.001). All types of social protection were more frequently reported among families with stunted children, such as the Family Hope Program (Program Keluarga Harapan) (8388, 26.0%), Non-Cash Food Assistance (Bantuan Pangan Non Tunai) (5999, 23.7%), Cooking Oil Cash Assistance (Bantuan Langsung Tunai Minyak Goreng) (2750, 24.5%), Village Fund Cash Assistance (Bantuan Langsung Tunai Dana Desa) (14,696, 25.4%), and Pre-Employment Program (Kartu Pra-kerja) (1870, 19.0%) (Supplementary material 3).

**Table 2:** Socio-demographic of malnutrition among relative middle-low wealth household

	Stunting		*P* value	Underweight		*P* value	Wasting		*P* value
	Yes (%)	No (%)		Yes (%)	No (%)		Yes (%)	No (%)	
Age									
0–5 month	1536 (0.5)	22,538 (7.8)	< 0.001	1184 (0.4)	22,890 (7.9)	< 0.001	1309 (0.5)	22,765 (7.9)	< 0.001
6–59 month	57,003 (19.7)	208,554 (72.0)		43,685 (15.1)	221,872 (76.6)		20,117 (6.9)	245,440 (84.7)	
Sex									
Male	31,740 (11.0)	116,837 (40.3)	< 0.001	23,763 (8.2)	124,814 (43.1)	< 0.001	12,135 (4.2)	136,442 (47.1)	< 0.001
Female	26,799 (9.3)	114,255 (39.4)		21,106 (7.3)	119,948 (41.4)		9291 (3.2)	131,763 (45.5)	
Wealth									
Poor	55,290 (19.1)	206,733 (71.4)	< 0.001	42,382 (14.6)	219,641 (75.8)	< 0.001	19,935 (6.9)	242,088 (83.6)	< 0.001
Middle	3249 (1.1)	24,359 (8.4)		2487 (0.9)	25,121 (8.7)		1491 (0.5)	26,117 (9.0)	
Regions type									
Urban	25,448 (8.8)	121,062 (41.8)	< 0.001	21,170 (7.3)	125,340 (43.3)	< 0.001	11,053 (3.8)	135,457 (46.8)	< 0.01
Rural	33,091 (11.4)	110,030 (38.0)		23,699 (8.2)	119,422 (41.2)		10,373 (3.6)	132,748 (45.8)	
Social protection ownership									
No	15,098 (5.2)	46,222 (16.0)	< 0.001	11,458 (4.0)	49,862 (17.2)	< 0.001	4977 (1.7)	56,343 (19.5)	< 0.001
Yes	43,441 (15.0)	184,870 (63.8)		33,411 (11.5)	194,900 (67.3)		16,449 (5.7)	211,862 (73.1)	
Cooking fuel									
Residual	12,403 (4.3)	34,313 (11.8)	< 0.001	9235 (3.2)	37,481 (12.9)	< 0.001	4466 (1.5)	42,250 (14.6)	< 0.001
Clean	46,136 (15.9)	196,779 (67.9)		35,634 (12.3)	207,281 (71.6)		16,960 (5.9)	225,955 (78.0)	
Drinking water									
Unimproved	23,482 (8.1)	90,835 (31.4)	< 0.001	18,612 (6.4)	95,705 (33.0)	< 0.001	9116 (3.1)	105,201 (36.3)	< 0.001
Improved	35,057 (12.1)	140,257 (48.4)		26,257 (9.1)	149,057 (51.5)		12,310 (4.3)	163,004 (56.3)	
Shared-toilet									
Yes	8084 (2.8)	19,834 (6.8)	< 0.001	5859 (2.0)	22,059 (7.6)	< 0.001	2362 (0.8)	25,556 (8.8)	< 0.001
No	50,455 (17.4)	211,258 (72.9)		39,010 (13.5)	222,703 (76.9)		19,064 (6.6)	242,649 (83.8)	

### Multivariable binary logistic regression analysis

Malnutrition status associated with a heightened risk of ARI and diarrhea. Among children, those classified as wasting demonstrated consistently the highest likelihood of developing diarrhea when contrasted with those experiencing stunting and underweight. Specifically, wasting children are 1.18 times (a*OR* = 1.18, 95% *CI* 1.05–1.19) more likely to be infected with diarrhea and 1.16 times (a*OR* = 1.16, 95% *CI* 1.09–1.24) more likely to suffer from ARI (Table 3). Furthermore, infants older than 6 months consistently exhibit protective factors against ARI and diarrhea compared to those younger than 6 months. Additionally, households that share toilet facilities are 1.45 times (a*OR* = 1.45, 95% *CI* 1.38–1.51) more at risk for diarrhea compared to households that do not share toilets.

**Table 3:** Multivariable binary logistic regression analysis of acute respiratory infection and diarrhea

Variables	Categories	Acute respiratory infection		Diarrhea	
		c*OR* (95% *CI*)	a*OR* (95% *CI*)	c*OR* (95% *CI*)	a*OR* (95% *CI*)
Age					
	0–5 month	1 (ref)	1 (ref)	1 (ref)	1 (ref)
	6–59 Month	0.48 (0.44–0.51)***	0.48 (0.45–0.52)***	0.33 (0.30–0.36)***	0.34 (0.31–0.37)***
Sex					
	Male	1 (ref)	1 (ref)	1 (ref)	1 (ref)
	Female	1.04 (1.01–1.07)**	1.03 (0.99–1.06)	1.13 (1.10–1.16)***	1.12 (1.09–1.16)***
Region type					
	Urban	1 (ref)	1 (ref)	1 (ref)	1 (ref)
	Rural	0.95 (0.93–0.98)**	1.03 (0.99–1.07)	0.95 (0.92–0.97)***	0.96 (0.93–0.99)*
Stunting					
	No	1 (ref)	1 (ref)	1 (ref)	1 (ref)
	Yes	1.20 (1.16–1.25)***	1.10 (1.05–1.15)***	1.25 (1.20–1.30)***	1.09 (1.05–1.14)***
Underweight					
	No	1 (ref)	1 (ref)	1 (ref)	1 (ref)
	Yes	1.18 (1.14–1.23)***	1.01 (0.96–1.07)	1.31 (1.26–1.36)***	1.11 (1.05–1.17)***
Wasting					
	No	1 (ref)	1 (ref)	1 (ref)	1 (ref)
	Yes	1.19 (1.12–1.25)***	1.18 (1.05–1.19)***	1.29 (1.22–1.36)***	1.16 (1.09–1.24)***
Social protection					
	Yes	1 (ref)	1 (ref)	1 (ref)	1 (ref)
	No	1.06 (1.02–1.10)**	1.02 (0.98–1.06)	1.21 (1.17–1.25)***	1.17 (1.13–1.22)***
Cooking fuel					
	Clean	1 (ref)	1 (ref)	1 (ref)	1 (ref)
	Residual	1.57 (1.51–1.63)***	1.53 (1.47–1.60)***	0.92 (0.88–0.96)***	0.84 (0.80–0.88)***
Drinking water					
	Improved	1 (ref)	1 (ref)	1 (ref)	1 (ref)
	Unimproved	1.07 (1.04–1.11)***	1.08 (1.04–1.11)***	1.04 (1.01–1.07)*	1.03 (1.01–1.07)*
Shared toilet					
	No	1 (ref)	1 (ref)	1 (ref)	1 (ref)
	Yes	1.32 (1.26–1.38)***	1.19 (1.13–1.25)***	1.46 (1.39–1.53)***	1.45 (1.38–1.51)***

c*OR*, Crude odd ratio; a*OR*, Adjusted odd ratio; *CI*, confidence interval; ref, reference; *** indicates *P* < 0.001; ** indicates* P* < 0.01; * indicates *P* < 0.05

Environmental factors such as households using residual cooking fuel, unimproved drinking water, and shared toilets also had higher risks for ARIs and diarrhea. Households using residual cooking fuel, unimproved drinking water, and shared toilets had risks of ARI infection of 1.53 (a*OR* = 1.53, 95% *CI* 1.47–1.60), 1.08 (a*OR* = 1.08, 95% *CI* 1.04–1.11), and 1.19 (a*OR* = 1.19, 95% *CI*: 1.13–1.25) times, respectively, compared to families using non-residual cooking fuel, improved drinking water, and non-shared toilets. Children lacking social protection consistently represent a risk factor for ARIs, and diarrhea. The risk for these children to contract diarrhea is 1.17 times (a*OR* = 1.17, 95% *CI* 1.13–1.22) higher compared to those with social protection.

## Discussion

This study consistently demonstrates that an age of over 6 months protects against Acute Respiratory Infections (ARI) and diarrhea. For ARI, the factors contributing to children under five years old vulnerability include age, stunting, wasting, unclean cooking fuel, unimproved drinking water, and shared toilet facilities. In the case of diarrhea, all identified variables exert an association. Notably, the most significant risk factors associated with ARI and diarrhea are unclean cooking fuel and shared toilet facilities, respectively. This finding highlights that the prevalence of ARI (5.7%) and diarrhea (6.0%) among households within the low-middle wealth index surpasses the national prevalence [5]. Research conducted in Cambodia and Philippines demonstrates that the prevalence of ARI and diarrhea is higher among poor families compared to affluent families [16, 27].

These findings found that those children aged under six months exhibit greater vulnerability to ARI, diarrhea, and malnutrition compared to older age groups. This observation is consistent with studies from economically disadvantaged areas in China and Kenya, which reveal a negative correlation between age and the risk of infection [28, 29]. The heightened susceptibility in infants under six months can be attributed to their still-developing respiratory, digestive, and immune systems, which rely on maternal antibodies [28]. Complementary feeding increases the risk of acute diarrhea in infants and can adversely affect children's health [28, 30]. Furthermore, introducing pre-lacteal feeding six months before may worsen this vulnerability, as the coverage of exclusive breastfeeding in Indonesia is inconsistent across regions [31]. A study conducted in Indonesia revealed that the prevalence of pre-lacteal feeding within the first three days of life among families classified as poorest and poor were 43.39% and 46.8%, respectively [24], specifically for honey (3.5%), and water (4.9%) as pre-lacteal feeding practices [32]. Improvements in maternal education at the population level are widely acknowledged as a crucial and extensively studied determinant of child health and survival [3]. Interventions that enhance mothers’ self-efficacy can bolster breastfeeding confidence and promote the continuation of breastfeeding practices until 6 months postpartum [33]. Such measures may act as a preventative strategy against pre-lacteal feeding in infants under 6 months, thereby playing a crucial role in reducing their susceptibility to ARI and diarrhea.

This study demonstrates an association between malnutrition issues and the past two-week prevalence of ARIs and diarrhea in low- and middle-wealth households. Earlier studies in Indonesia have indicated that lower household wealth levels correlate with a heightened risk of malnutrition and ARIs [11, 12]. A study in Philippines reported that children from lower economic families was more frequently reported compared with high economic families (79.87% vs. 20.13%) [27]. The short-term effects of this issue on impoverished families include a weakened immune system, a higher risk of developing ARI and diarrhea, as well as delays in motor skills and cognitive and social development during childhood [34]. Research in Indonesia also reported that the poorest districts exhibited a significantly higher prevalence of underweight, wasting, and stunting than the wealthiest districts [35]. This aligns with findings indicating that malnutrition issues and morbidity distribution is more prevalent outside of Java and Bali, which are closely associated with areas of high wealth index [36]. Limited access to healthcare services and proper sanitation facilities outside of Java and Bali complicates malnutrition and morbidity issues for low- and middle-wealth households, making them increasingly complex and challenging to address [37].

This study emphasizes that inadequate household sanitation support can increase the risk of ARIs and diarrhea among children. The findings of this study indicate that 16.1% of households utilize unclean cooking fuel, with 1.3% suffering from ARI. Those using unclean cooking fuel have a 1.57 times greater risk of ARI infection. Regarding diarrhea, 9.7% of households with shared toilet facilities report cases, with 0.8% affected, and individuals in these households have a 1.45 times higher risk of experiencing diarrhea. These results align with other national-level studies in the Philippines, Cambodia and Myanmar that reported lack of sanitation and cooking fuel among low-middle wealth household might increase the risk of ARIs and diarrhea [16, 27, 38]. Indonesia’s achievements in reducing the use of residual cooking fuels, coupled with the support of social protection programs, should be expanded to further enhance the health outcomes of children under five. These efforts could also be adapted and scaled within the broader Southeast Asian region. This study found that social protection support is vital in preventing ARI and diarrhea issues among families with low to middle-wealth status. This study underscores the risks of ARIs, diarrhea, and malnutrition in groups lacking social protection guarantees. The conversion of clean cooking fuel in Indonesia, aimed at reduction of indoor pollution in low- and middle-income households [39] while Social protection policies and programs can offer a crucial safety net for children and caregivers from vulnerable households by alleviating poverty and addressing the underlying determinants of malnutrition [40]. Furthermore, research in Indonesia indicates that the food voucher program (Bantuan Pangan Non Tunai) increased dietary diversity among poor households by at least 15 percentage points compared to those that continued to receive in-kind food transfers. Additionally, this new initiative has enhanced the consumption of essential nutrients among impoverished households and improved the targeting effectiveness of social welfare programs [41]. Indonesia has successfully strengthened its social protection system for low- and middle-income communities in addressing the challenges posed by the COVID-19 pandemic. The rice in-kind transfer program (“Rastra”) was gradually replaced by a food voucher program (“Sembako”), which aims to diversify diets among vulnerable households. [42]. The combination of social protection systems in Indonesia is crucial in giving households the flexibility to choose food according to their preferences [41]. Strengthening and developing the implementation of pro-poor health and social program policies is essential to enhance the participation of low- to middle-wealth index households in accessing healthcare and education, including preventing infectious diseases in young children.

While the present study has primarily focused on socioeconomic and environmental determinants of childhood diarrhea, it is imperative to acknowledge that co-infections with multiple enteric pathogens may also constitute a critical, yet underexplored, component of the disease’s etiology. Although our analysis did not encompass this dimension, extant evidence suggests that the interplay between bacterial and viral pathogens can synergistically exacerbate disease severity, particularly in settings characterized by inadequate sanitation [6, 43]. This observation underscores the necessity for integrated sanitation and infection control strategies that concurrently address both bacterial and viral transmission routes [44]. Emphasizing this aspect is essential for guiding the design and implementation of differentiated targeted preventive measures aimed at mitigating the overall burden of diarrheal disease in vulnerable populations.

This study employs a large sample size that encompasses nearly all children under five across various districts and cities in Indonesia nationally; however, it faces certain research limitations. First, although the principal component analysis approach has been widely used in other studies to measure household wealth levels, its application has inherent constraints within this context [45]. However, the household wealth index in this study heavily relies on the selected parameters, and not all the parameters adequately reflect the family’s welfare conditions. The validity of this approach should be further tested with different study designs and parameters. Second, this study employs a cross-sectional approach, which limits the ability to draw causal conclusions over time. Third, there is a potential for bias, as parent respondents may misremember or forget conditions from the two weeks before the interview, particularly regarding clinical symptoms of ARI and diarrhea in their children. Additionally, limited access to healthcare services in Indonesia may lead to undiagnosed cases of ARI and diarrhea, potentially affecting the research outcomes. Despite these limitations, this study provides crucial information regarding the risk factors for ARI and diarrhea among Indonesia’s low- and middle-income households. Furthermore, it is the first to report on the impact of social protection on malnutrition issues and the incidence of ARI and diarrhea in children under five from low- to middle-wealth families. This can assist the government in strengthening pro-poor implementation policies through the social protection system. The results provide a basis for future research and support the design and implementation of targeted prevention strategies for ARIs and diarrhea, as well as informing poverty reduction programs and addressing health challenges among children health-related SDGs.

## Conclusions

This study highlights the need for targeted interventions to address malnutrition, poor sanitation, and limited social protection, which contribute to the high rates of ARI and diarrhea among children under five in low- and middle-income households in Indonesia. Recommendations include strengthening social protection programs, improving access to clean water and sanitation, and advocating for policies that reduce poverty and enhance income support through direct transfers and child benefits, particularly in vulnerable communities.

## Supplementary Information

Additional file 1

## Data Availability

The dataset used in this study was acquired from a third-party source and is not publicly available. Disclosure of the data is restricted by the data provider and the Ministry of Health of the Republic of Indonesia, which holds the ownership rights. Researchers seeking access to the Indonesian National Nutritional Status Survey 2022 dataset may request it through https://layanandata.kemkes.go.id/, though access to sensitive information is limited to those who fulfill the required eligibility criteria..

## Abbreviations

ARI: Acute respiratory infection
a*OR*: Adjusted odd ratio
*CI*: Confidence interval
HA*Z*: Height-for-age *Z* score
IMCI: Integrated management of childhood illness
VIF: Variance inflation factor
WA*Z*: Weight-for-age *Z* score
WHO: World Health Organization
WH*Z*: Weight-for-height *Z* score
