# Supplementary material for: Prevalence and risk factors of acute respiratory infection and diarrhea among children under 5 years old in low-middle wealth household, Indonesia
Source: Infect Dis Poverty. 2025 Feb 27;14:13. doi: 10.1186/s40249-025-01286-9 (PMC11866638; doi:10.1186/s40249-025-01286-9)
Supplement: Supplementary file 1 — Additional file 1 [file 40249_2025_1286_MOESM1_ESM.docx]

**Supplementary material 1. Correlation coefficient matrix of variables**

|  | Stunting | Underweight | Wasted | Social Protection | Cooking Fuel | Shared Toilet | Drinking Water | Region Type | Sex | Age |
| --- | --- | --- | --- | --- | --- | --- | --- | --- | --- | --- |
| Stunting | 1.000 | -0.506 | 0.197 | -0.032 | -0.042 | -0.043 | -0.007 | 0.051 | -0.033 | 0.053 |
| Underweight | -0.506 | 1.000 | -0.486 | -0.017 | -0.005 | -0.014 | -0.009 | -0.004 | 0.015 | 0.031 |
| Wasted | 0.197 | -0.486 | 1.000 | 0.000 | -0.029 | 0.004 | -0.011 | -0.013 | -0.034 | -0.004 |
| Social Protection | -0.032 | -0.017 | 0.000 | 1.000 | -0.018 | -0.058 | 0.021 | 0.042 | 0.004 | 0.002 |
| Cooking Fuel | -0.042 | -0.005 | -0.029 | -0.018 | 1.000 | -0.157 | 0.022 | 0.177 | 0.000 | -0.009 |
| Shared Toilet | -0.043 | -0.014 | 0.004 | -0.058 | -0.157 | 1.000 | -0.024 | 0.098 | -0.002 | -0.004 |
| Drinking Water | -0.007 | -0.009 | -0.011 | 0.021 | 0.022 | -0.024 | 1.000 | -0.090 | -0.001 | -0.001 |
| Region Type | 0.051 | -0.004 | -0.013 | 0.042 | 0.177 | 0.098 | -0.090 | 1.000 | 0.002 | 0.005 |
| Sex | -0.033 | 0.015 | -0.034 | 0.004 | 0.000 | -0.002 | -0.001 | 0.002 | 1.000 | -0.003 |
| Age | 0.053 | 0.031 | -0.004 | 0.002 | -0.009 | -0.004 | -0.001 | 0.005 | -0.003 | 1.000 |

**Supplementary material 2. Multicollinearity of independent variables**

| Variable | Variance Inflation Factor |
| --- | --- |
| Stunting | 1.38 |
| Underweight | 1.69 |
| Wasted | 1.30 |
| Social Protection | 1.01 |
| Cooking Fuel | 1.06 |
| Shared Toilet | 1.04 |
| Drinking Water | 1.01 |
| Region Type | 1.06 |
| Sex | 1.00 |
| Age | 1.01 |

**Supplementary material 3 The geographic distribution of acute respiratory infection (A) and diarrhea (B) among children under 5 years old from low-and middle- wealth household in Indonesia. Darker shades of black signify a higher proportion of acute respiratory infection and diarrhea in these areas.**

**
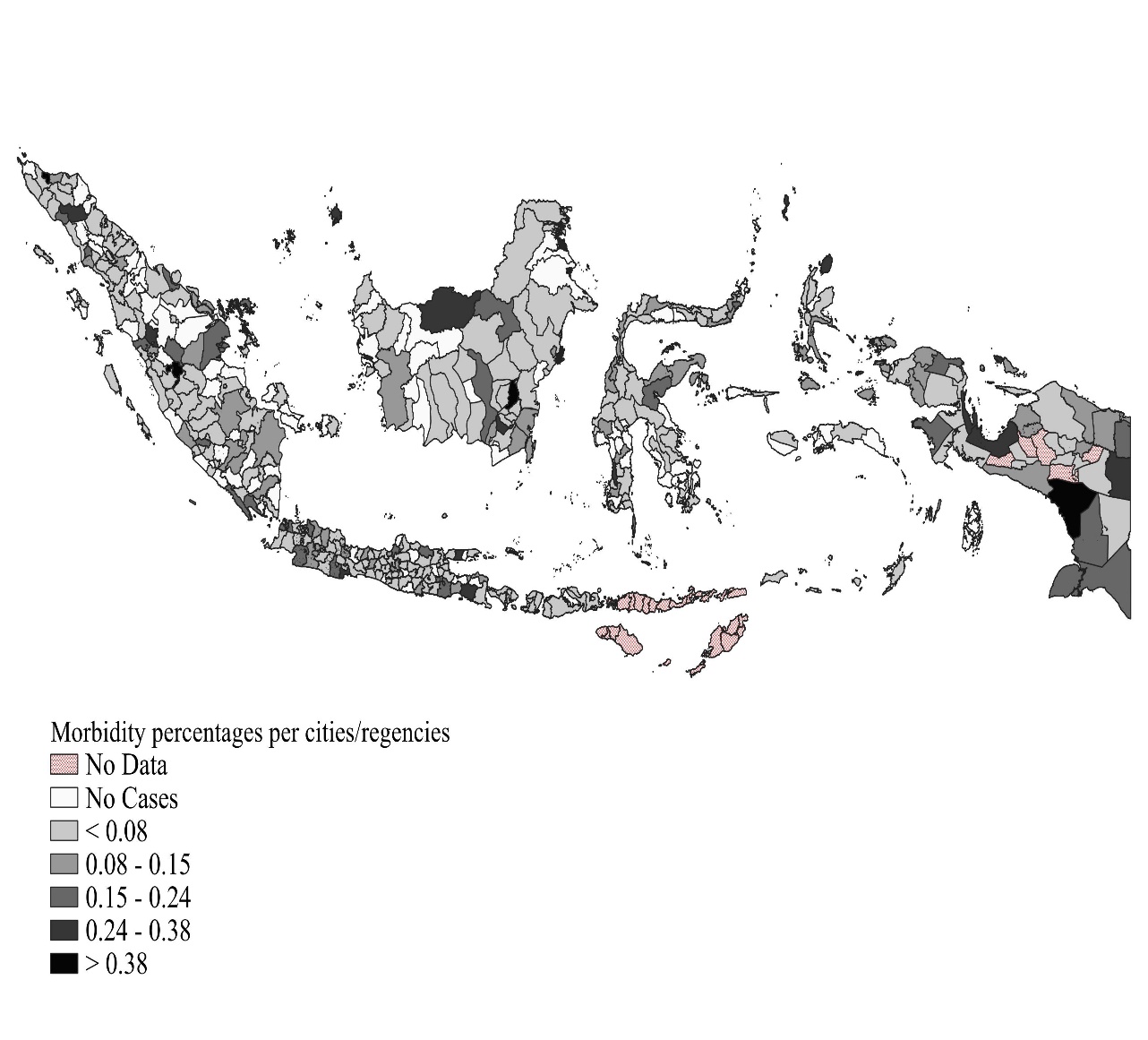

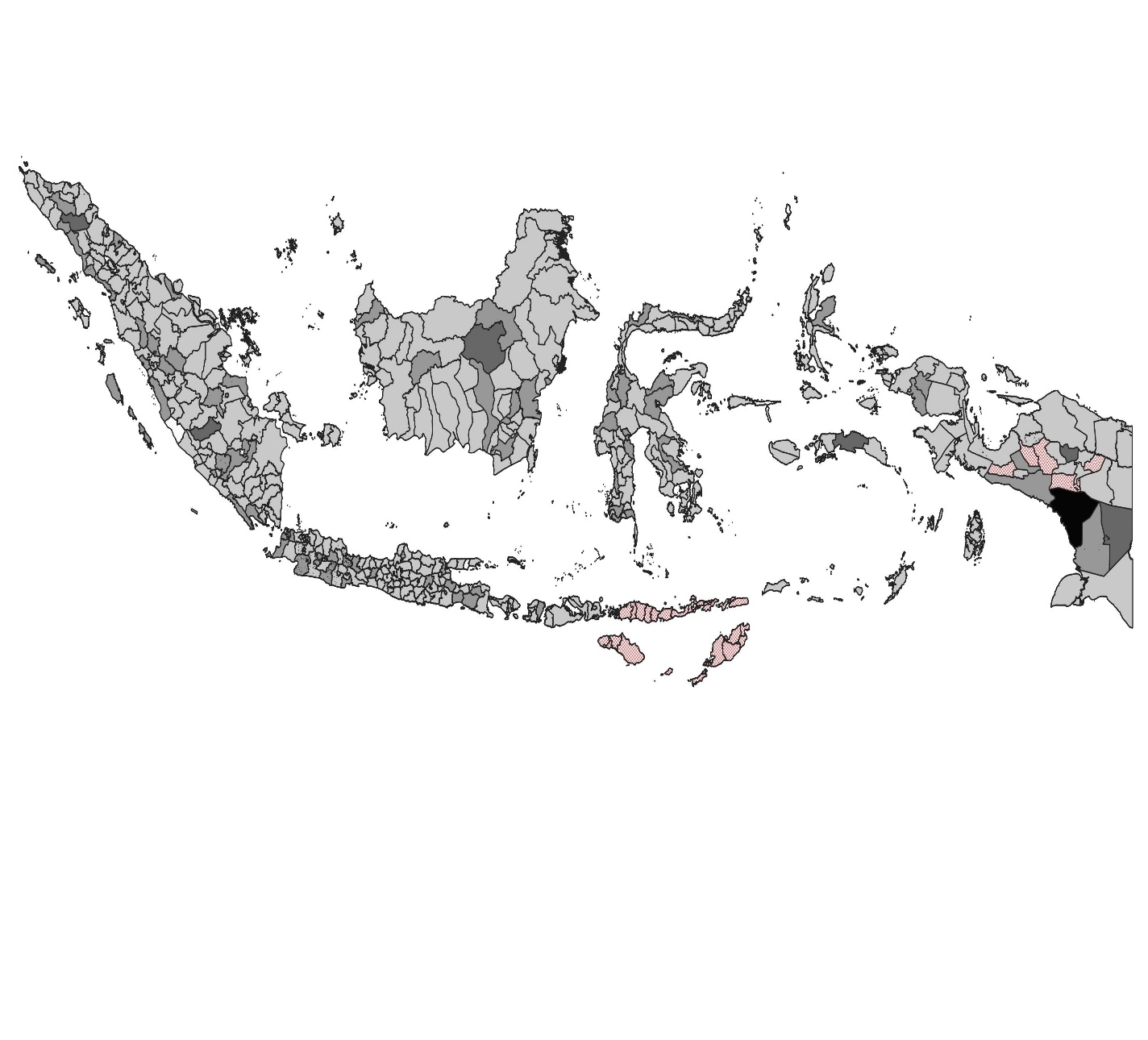

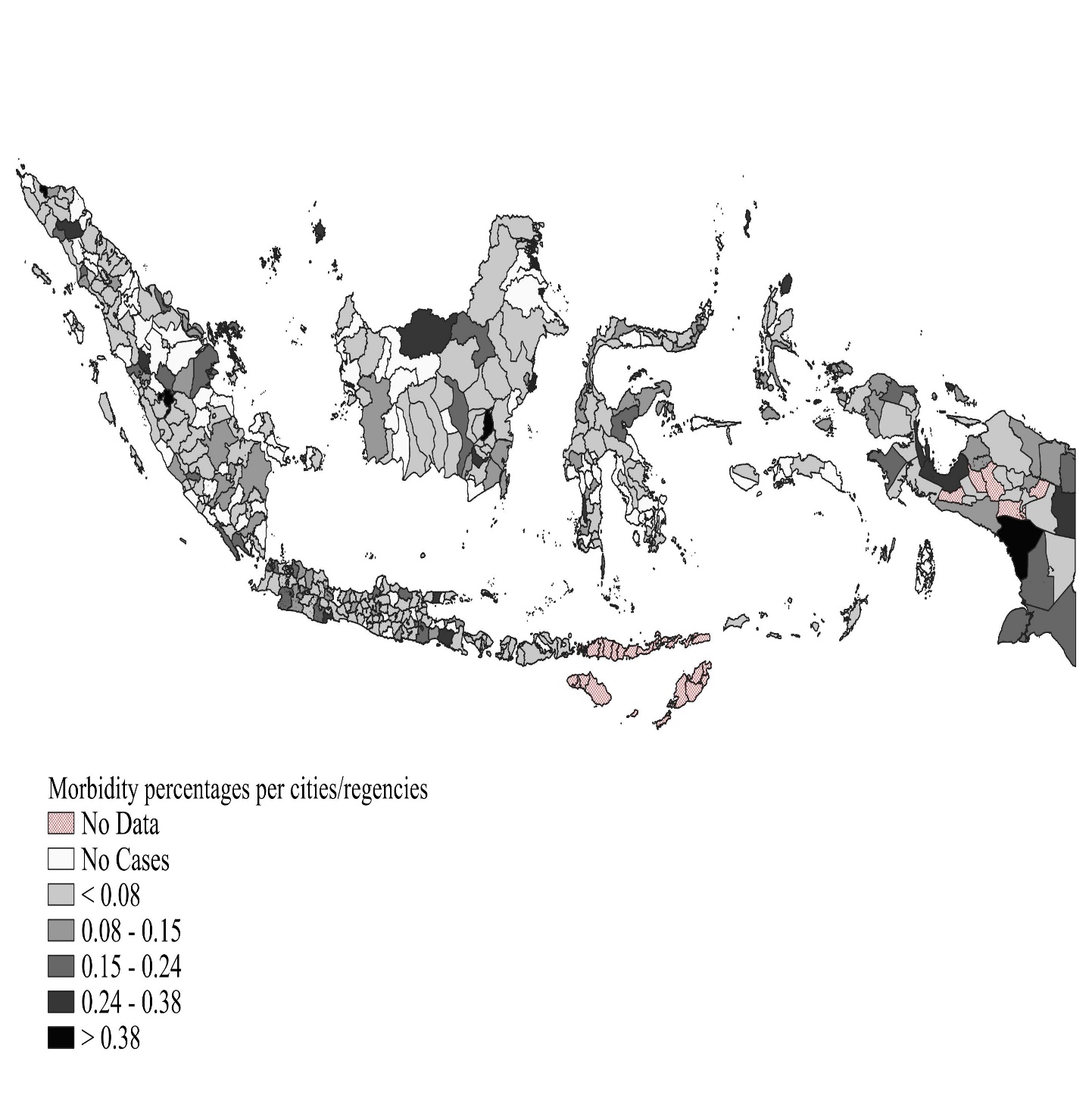
**

**Supplementary material 3. Type of social protection**

|  | ARIs (%) | Diarrhea (%) | Stunting (%) | Underweight (%) | Wasting (%) |
| --- | --- | --- | --- | --- | --- |
| The Family Hope Program (Program Keluarga Harapan) (n=32,248) | 1877 (5.8) | 2220 (6.9) | 8388 (26.0) | 6291 (19.5) | 2605 (8.1) |
| Non-Cash Food Assistance (Bantuan Pangan Non Tunai) (n=25,342) | 1389 (5.5) | 1762 (7.0) | 5999 (23.7) | 4617 (18.2) | 1985 (7.8) |
| Cooking Oil Cash Assistance (Bantuan Langsung Tunai Minyak Goreng) (n=11,231) | 652 (5.8) | 835 (7.4) | 2750 (24.5) | 2123 (18.9) | 930 (8.3) |
| Village Fund Cash Assistance (Bantuan Langsung Tunai Dana Desa) (n=57,809) | 4188 (7.2) | 4085 (7.0) | 14696 (25.4) | 10,566 (18.3) | 4716 (8.2) |
| Pre-Employment Program (Pra Kerja) (n=9,851) | 560 (5.7) | 752 (7.6) | 1870 (19.0) | 1511 (15.3) | 737 (7.5) |
